# Delayed Diagnosis of Scabies: A Case Report

**DOI:** 10.7759/cureus.107569

**Published:** 2026-04-23

**Authors:** Kiley Hassevoort, Peyton Grant, Ava Thielman, Rachel Delost

**Affiliations:** 1 Dermatology, A.T. Still University Kirksville College of Osteopathic Medicine, Kirksville, USA; 2 Anatomy, A.T. Still University Kirksville College of Osteopathic Medicine, Kirksville, USA; 3 Medicine, A.T. Still University Kirksville College of Osteopathic Medicine, Kirksville, USA; 4 Dermatolgy, Optima/Advanced Dermatology, Cleveland, USA

**Keywords:** clinical presentation of scabies, crusted scabies, diagnose, diagnosis of scabies, : norwegian scabies

## Abstract

Classical scabies is a cutaneous disease characterized by an intense, pruritic rash. The scabies mite, *Sarcoptes scabiei*, localizes to skin-fold areas of the body. Crusted scabies is a manifestation of the disease, more common in immunocompromised individuals, and has been observed following systemic therapeutic medications that weaken the immune system. The disease leads to uncontrolled mite proliferation in the skin, characterized by areas of plaques with a parakeratotic crust. Crusted scabies is rare, with limited cases reported in the literature and, consequently, often associated with a delayed diagnosis and worsening of the condition. This case report discusses a 70-year-old male presenting with a two-year history of a pruritic rash on the neck, trunk, arms, and legs, which later progressed to hyperkeratotic plaques and was diagnosed as crusted scabies. This case underscores the importance of a thorough physical examination and a broad differential diagnosis, and consideration of scabies, especially in pruritic rashes unresponsive to various therapies.

## Introduction

Scabies is caused by the scabies mite called *Sarcoptes scabiei*. The scabies mite burrows in the stratum corneum, causing a humoral and delayed hypersensitivity reaction. Classical scabies, or scabies simplex, is characterized by intense itching with lesions most commonly located on the webs of fingers, wrists, palms, elbows, axilla, lower buttocks, inner thighs, waists, and soles of the feet [1]. Crusted scabies is a rare manifestation of scabies characterized by localized horny plaques, consisting of a parakeratotic crust that can vary in color, such as creamy, grey, yellow-brown, or yellow-green [1,2]. The pathogenesis of the crusted variant of scabies may be related to elevated interleukin-4 levels, leading to uncontrolled mite proliferation in the skin [2]. Scabies transmission occurs through skin-to-skin contact. Risk factors for this include sleeping next to an infested person, sexual contact, sharing living spaces, or close continuous contact with those infested [1,2]. Crusted scabies can be complicated by secondary bacterial infection from *Staphylococcus aureus*, leading to impetigo, ecthyma, cellulitis, and lymphangitis [2].

The diagnosis of scabies is based on clinical findings and the presence of the mite. Currently, no gold-standard diagnostic technique for scabies has been established, leading to frequent misdiagnosis. Diagnostic tools include microscopic examination of the skin scrapings on a glass slide with mineral oil, dermoscopy, and histopathology. However, the low sensitivity of these tests can cause a delay in diagnosis. Scabies often mimics other dermatological conditions, further delaying the diagnosis. The differential diagnosis for scabies includes psoriasis, eczema, seborrheic dermatitis, lichen planus, and cutaneous lymphoma [2]. This case highlights the adverse effects of a delayed diagnosis of scabies and the unnecessary administration of multiple topical and systemic therapies, leading to the worsening of the condition by the development of crusted scabies.

## Case presentation

**Figure 1:**
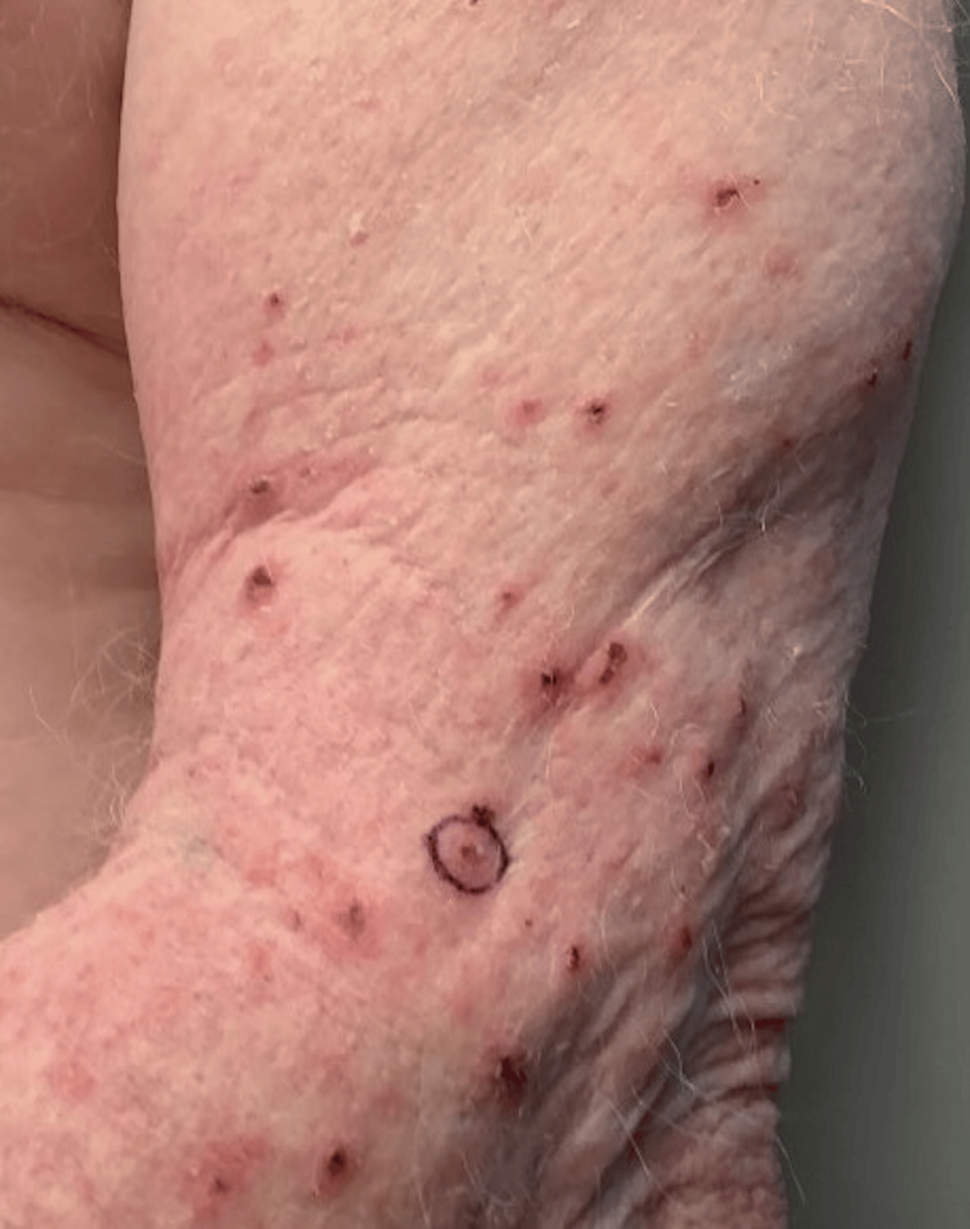
Left Arm The circled area represents the biopsy site, with erythematous papules and excoriations scattered throughout the arm.

A 70-year-old otherwise healthy male presented with a two-year history of a pruritic rash on the neck, trunk, arms, and legs. He denied any recent travel or any new personal care products. He lives with his wife, who denied any rashes or itching, and she did not have any signs or symptoms of infestation with scabies at any point. He did not have any household pets. The patient is a pastor and is in contact with many people day to day. He was unable to recall any contacts presenting with similar symptoms and denied any prolonged or close physical contact with anyone. During his initial visit, the physical examination revealed erythematous papules and areas of excoriations (Figure 1).

Dermoscopy was also used to further examine the papules and excoriations, revealing white and brown scales with patchy erythema. The patient’s workup included punch biopsies on the arm and leg, as well as a skin scraping with microscopic examination. The microscopic examination results were insignificant. The pathology report from these biopsies resulted in chronic actinic and spongiotic dermatitis, pointing to an initial diagnosis of dermatitis unspecified. When considering this pathology result and the diagnostic differential, the chronic actinic dermatitis result was consistent with the patient’s history of extensive sun exposure, emphasizing his diffuse actinic damage and his clinical findings, as seen in Figure 1.

**Figure 2:**
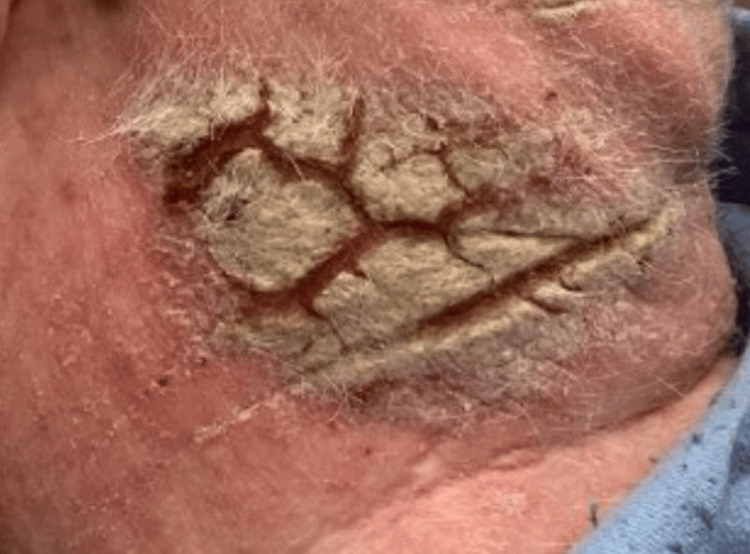
Posterior Neck An erythematous, eczematous, and lichenified plaque located on the posterior neck.

**Figure 3:**
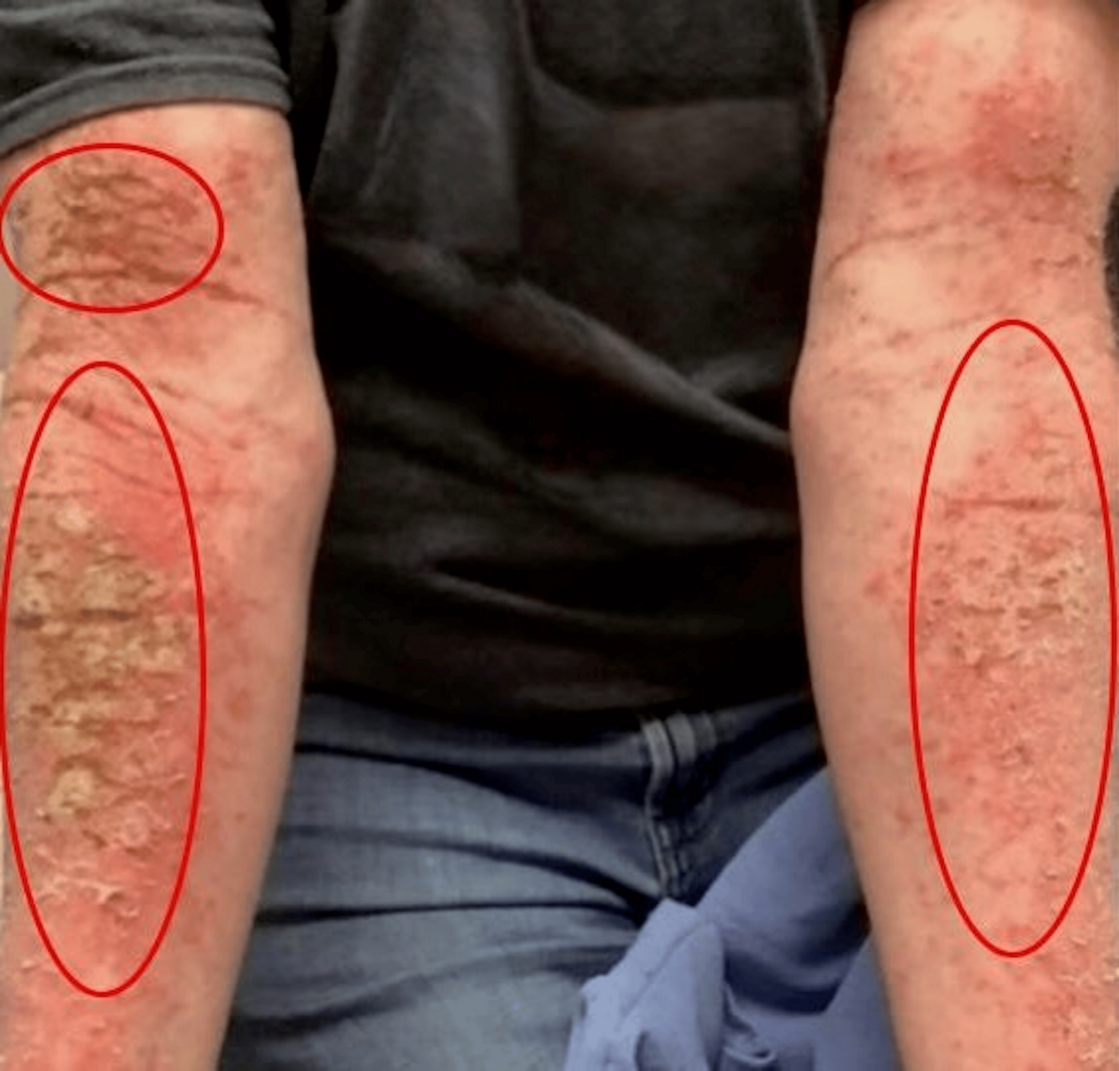
Upper Extremities The red circles highlight the erythematous, eczematous, and lichenified plaques along the upper extremities, though the lesions are scattered throughout.

**Figure 4:**
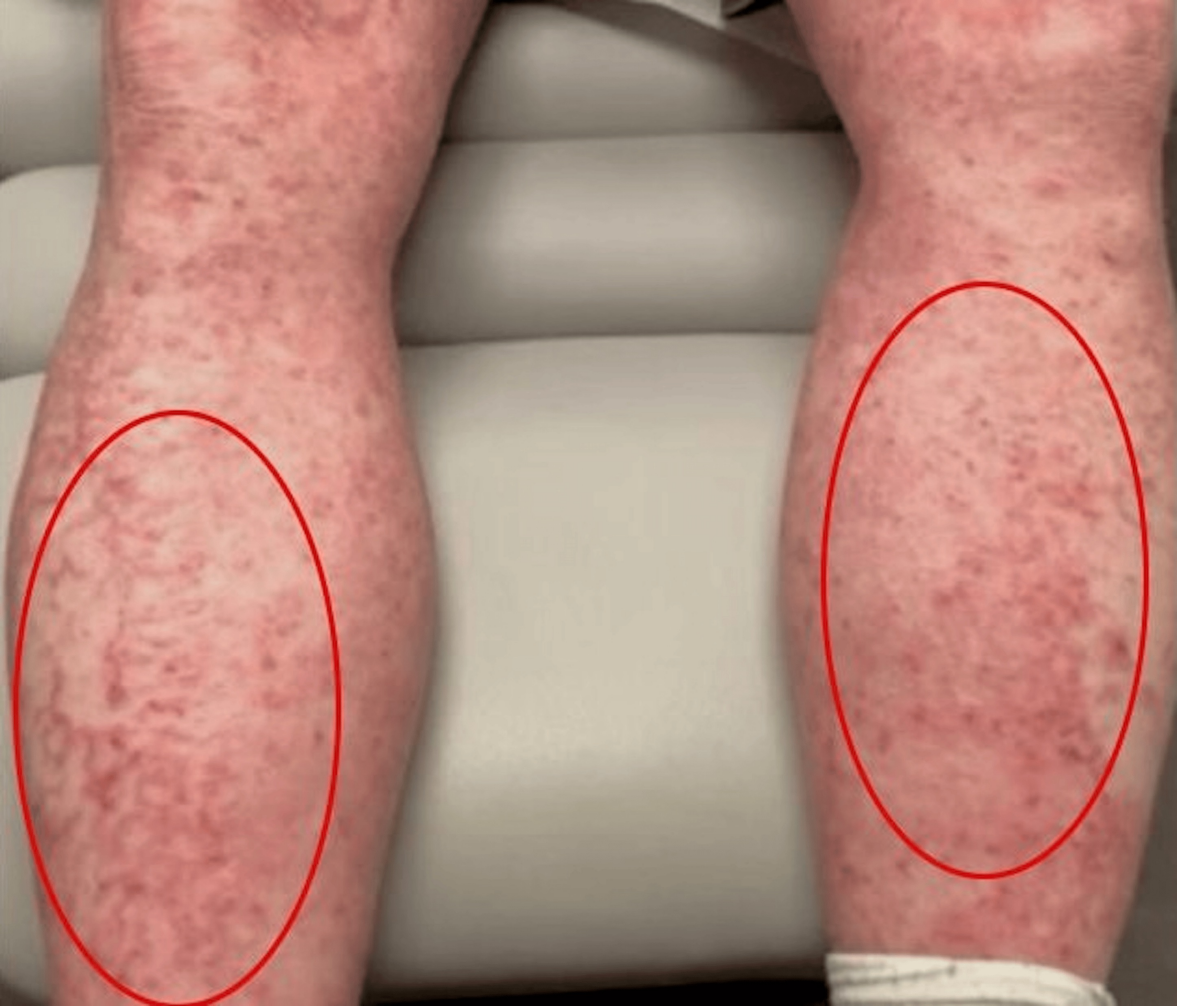
Lower Extremities The red circles emphasize the erythematous, eczematous, and lichenified plaques along the lower extremities, though the lesions are scattered throughout.

Given the initial diagnosis of dermatitis unspecified and attempting to resolve this patient’s pruritus, he tried and failed numerous therapies, including topical steroids, topical calcineurin inhibitors, prednisone, upadacitinib, abrocitinib, dupilumab, mycophenolate mofetil, and guselkumab throughout the course of a year. These treatment options improved the symptoms; however, the rash persisted and eventually progressed to erythematous, eczematous, and lichenified plaques (Figures 2-4).

**Figure 5:**
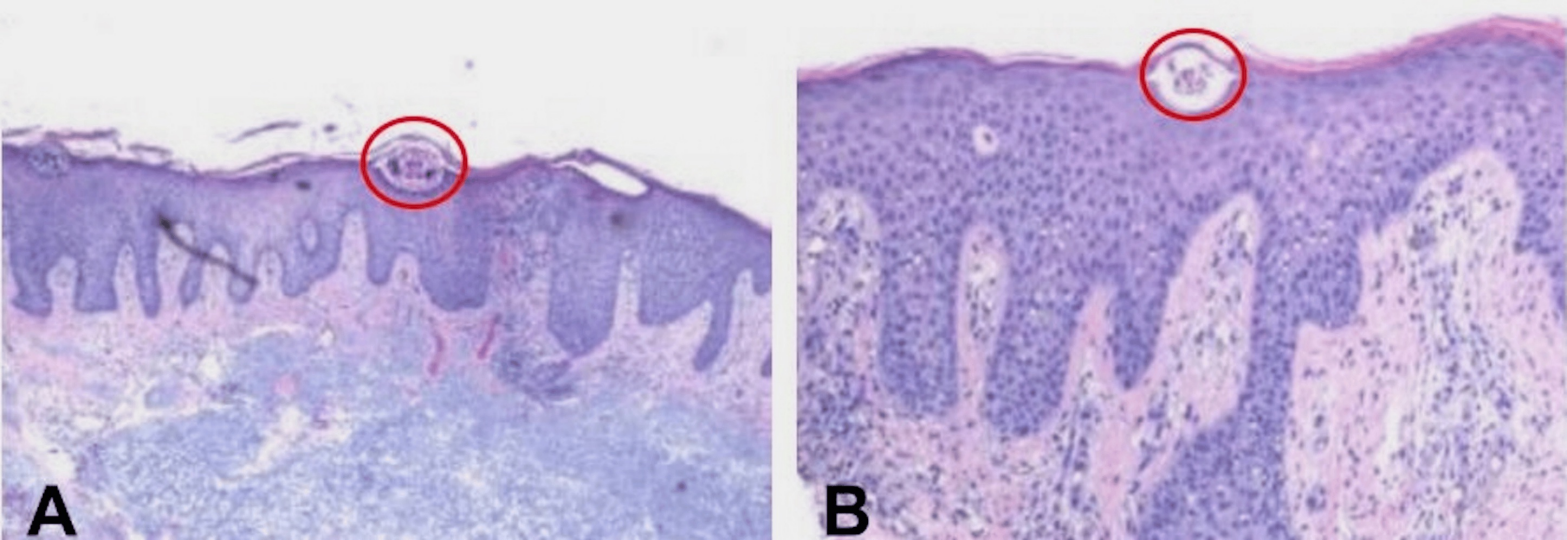
Photomicrographs From Biopsies Obtained From Right Forearm (A) and Right Thigh (B) Hematoxylin and eosin stain revealing burrows consisting of spaces between the stratum corneum and underlying keratinocytes, consistent with a scabies mite. Both photomicrographs demonstrate an underlying perivascular and interstitial mixed cell infiltrate of lymphocytes, eosinophils, and histiocytes. Original magnification is 4x for (A) and 10x for (B). The red circles highlight the locations of mite burrows.

With this new presentation of his condition, new biopsies were performed on the right forearm and right thigh. The pathology report from these biopsies finally revealed burrows, consisting of spaces between the stratum corneum and underlying keratinocytes, with mites found within them, leading to a final diagnosis of crusted scabies (Figure 5A,5B). The patient was then treated with oral ivermectin and topical permethrin, and the patient’s condition eventually resolved.

## Discussion

Scabies is a cutaneous disorder characterized by pruritic rash, while crusted scabies is characterized by nonpruritic hyperkeratosis of the skin due to uncontrolled proliferation of the scabies mite. Since crusted scabies results from the immune system's failure to control scabies mite proliferation, it is more commonly seen in immunocompromised individuals; however, it should not be ruled out in immunocompetent individuals [1,2]. A case was reported of a 30-year-old patient with no comorbid conditions who presented a three-month history of a generalized skin eruption before developing the hyperkeratotic lesion of crusted scabies [3]. Similar to our patient, the 30-year-old patient in the prior case report was misdiagnosed and treated with no success [3]. This signifies the significance of scabies mimicking other dermatologic conditions, making diagnosis more challenging. This case further emphasizes the importance of a broad differential diagnosis and a high index of suspicion even in an immunocompetent patient presenting with a non-specific rash and pruritus.

Scabies transmission typically occurs through skin-to-skin contact. Risk factors for this include sleeping next to an infected person, sexual contact, sharing living spaces, or close continuous contact with those infested [1,2]. This patient did not report any of these significant risk factors that contributed to contracting scabies. His wife remained asymptomatic throughout this patient’s clinical course. Although he interacts with many individuals in his role as a pastor, he denied any prolonged or close physical contact with others. The source of transmission in this case remains unclear.

Diagnosing scabies can be challenging and often misdiagnosed, particularly because standardized diagnostic methods are lacking and have low sensitivity levels. The diagnosis of scabies is based on clinical findings, patient history, and the demonstration of the mite. Various diagnostic tools are used to help determine the presence of the scabies mite. One way is to examine a skin scraping on a glass slide under a microscope with mineral oil [1,2]. This can reveal mites, eggs, and mite feces (scybala). Although the microscopy of skin scrapings is highly specific, its sensitivity is low, and it is operator-dependent. Biopsies are another way to diagnose scabies by revealing a burrow(s) with a mite or scybala on histopathology [1,2]. However, if a burrow or mite is absent on either biopsy or skin scraping, the diagnosis may be missed, and scabies cannot be ruled out.

Dermoscopy with polarized or ultraviolet (UV) light can also be useful for detecting mites. Under polarized light, the delta sign, or “hang glider” sign, is used to describe the shape of the mite's head. In UV mode, the mite produces a bright white reflection and appears as an oval-shaped reef representing the entire mite [4,5]. Tunnel borders can also be visualized under both polarized and UV modes [5]. Although dermoscopy is a rapid tool for detecting the mite and/or its burrow, it is expensive, and its sensitivity ranges from 15% to 90% depending on the user’s expertise [6].

According to Iyengar et al., the International Alliance for the Control of Scabies developed criteria using the Delphi approach to diagnose scabies. This criterion incorporates three levels of diagnostic certainty. Level A is confirmed scabies, which requires visualization of the mite or its products [4]. Level B is clinical scabies, and Level C is suspected scabies, which relies on clinical assessment of signs and symptoms, incorporating noticeable features on exam and history [4]. Levels B and C are made only if other differentials are less likely; however, this can still lead to a misdiagnosis of scabies, prolonging appropriate treatment.

This patient’s combination of an insignificant initial pathology report and negative skin scraping pointed to the diagnosis of dermatitis unspecified and skin damage from chronic sun exposure. The nonspecific diagnosis led to the use of unnecessary topical and systemic therapies in an immunocompetent patient for symptomatic management, which further compromised his immune system and promoted disease progression to crusted scabies. Two notable cases of crusted scabies have been reported after the use of corticosteroids in both an adult and an infant, correlating with this patient’s clinical course [7,8]. A systematic review further supports that the use of immunosuppressive therapies, especially corticosteroids, is among the highest risk factors for crusted scabies [9]. These studies support the hypothesis that this patient’s use of systemic therapies weakened his immune system, exacerbating his classical scabies and allowing for the escalation to crusted scabies. This highlights the role of iatrogenic immunosuppression after using systemic therapies that targeted the patient’s symptoms after insignificant biopsy and microscopy findings, as well as the limited and low sensitivity of the diagnostic tools currently available.

The recommended treatment for scabies includes either permethrin cream, oral ivermectin, or benzyl benzoate. Treatment for crusted scabies includes both a topical scabicide, such as permethrin cream or benzyl benzoate lotion, and oral ivermectin [10]. In addition, patients' clothing, bedding, and towels should be cleaned [10]. This case further supports the use of oral ivermectin for scabies and its effectiveness, as the patient’s scabies resolved after treatment with ivermectin.

## Conclusions

In this report, we describe a case of an immunocompetent man diagnosed with crusted scabies approximately one year after presenting with symptoms that were misdiagnosed as dermatitis unspecified and managed with inappropriate treatment. The nonspecific findings on punch biopsy and skin scraping resulted in a delayed diagnosis and administration of inappropriate pharmacotherapies that may have weakened his immune system and contributed to disease progression. This case highlights the resultant iatrogenic immunosuppression that can occur while chasing the "itch" and the limited sensitivity of diagnostic testing. Improved diagnostic methods for scabies are critical for reducing poor outcomes, including inappropriate therapy, extended clinical course, and disease progression to crusted scabies.
